# Overexpression of *llm1* Affects the Synthesis of Secondary Metabolites of *Aspergillus cristatus*

**DOI:** 10.3390/microorganisms10091707

**Published:** 2022-08-24

**Authors:** Yunsheng Wang, Yincui Chen, Jin Zhang, Chuanbo Zhang

**Affiliations:** School of Life Sciences, Guizhou Normal University, Huaxi University Town, Gui’an New District, Guiyang 550025, China

**Keywords:** *Aspergillus cristatus*, *llm1* gene, overexpression, secondary metabolite, RNA-Seq

## Abstract

Putative methyltransferases are thought to be involved in the regulation of secondary metabolites in filamentous fungi. Here, we report the effects of overexpression of a predicted *LaeA*-like methyltransferase gene *llm1* on the synthesis of secondary metabolites in *Aspergillus cristatus.* Our results revealed that overexpression of the gene *llm1* in *A. cristatus* significantly hindered the production of conidia and enhanced sexual development, and reduced oxidative tolerance to hydrogen peroxide. Compared with the wild-type, the metabolic profile of the overexpression transformant was distinct, and the contents of multiple secondary metabolites were markedly increased, mainly including terpenoids and flavonoids, such as (S)-olEuropeic acid, gibberellin A62, gibberellin A95, ovalitenone, PD 98059, and 1-isomangostin. A total of 600 significantly differentially expressed genes (DEGs) were identified utilizing transcriptome sequencing, and the DEGs were predominantly enriched in transmembrane transport and secondary metabolism-related biological processes. In summary, the strategy of overexpressing global secondary metabolite regulators successfully activated the expression of secondary metabolite gene clusters, and the numerous secondary metabolites were greatly strengthened in *A. cristatus*. This study provides new insights into the in-depth exploitation and utilization of novel secondary metabolites of *A. cristatus*.

## 1. Introduction

Fu-brick tea (FBT) is a traditional post-fermented tea with various health care effects [1,2,3]. *Aspergillus cristatus* is the dominant microbe during the maturation process of FBT, and has a huge role in the unique taste, color, and pharmacology of the tea; furthermore, the quantity of *A. cristatus* is critical for evaluating the quality of FBT [4,5]. As a probiotic, *A. cristatus* is instrumental in the transformation of raw material components and the production of various secondary metabolites during fermentation to improve the quality and efficacy of fermented products [6,7,8], which highlight the significant application value and prospects of *A. cristatus*. A total of 39 gene clusters for the synthesis of secondary metabolites were predicted to be encoded in the genome of *A. cristatus* [9], which indicates the great potential of *A. cristatus* to synthesize secondary metabolites. However, the gene clusters for secondary metabolites encoded in filamentous fungal genomes are not expressed or exhibit very low expression levels under routine laboratory culture [10,11,12], which markedly hinders the utilization of *A. cristatus* as a source of secondary metabolites.

The method of overexpression or knockout of global secondary metabolite regulators is widely used to fully explore the secondary metabolite potential of fungi. *laeA* is one of the examples of secondary metabolite regulators with a conserved methyltransferase domain, and was first identified by Bok and colleagues [13]. Overexpression of *laeA* in *Monascus purpureus* increased the production of the lipid-lowering secondary metabolite lovastatin [14]. Similarly, overexpression of *laeA* in other filamentous fungi increased the content of secondary metabolites [15,16].

Further research identified putative methyltransferases in fungal genomes that were similar to *laeA*, and these were named *laeA*-like methyltransferases. In *Aspergillus nidulans*, these genes were named *llmA–llmJ* [17]. Knockdown of *llmF* reduced the production of sterigmatocystin, and *llmF* showed positive regulation of conidiation. Nine genes encoding *laeA*-like methyltransferases (*llm1-9*) were also identified in the *Cochliobolus heterostrophus* genome [18], and knockdown of *llm1* resulted in improved T-toxin production, indicating the *llm1* negative regulation of the production of this toxin. However, overexpression of a *laeA*-like gene (*llmG*) in *A. nidulans* led to increased production of sterigmatocystin [19]. Overexpression of the *laeA*-like gene in *Daldinia eschscholzii* also resulted in the production of two novel metabolites [20]. These indicated that *laeA*-like methyltransferases are similarly involved in the regulation of secondary metabolites and development in filamentous fungi, and that the regulatory pattern varies among species.

Although *laeA*-like methyltransferases are well studied in specific species of *Aspergillus* sp., there are few relevant reports regarding *A. cristatus*. Consequently, this study aimed to address this gap, and a *laeA*-like methyltransferase (Llm1 encoded by *llm1*) was identified in *A. cristatus*. We further evaluated the activation of silent secondary metabolite pathways via overexpression of *llm1* gene of *A. cristatus* to provide guidance for novel secondary metabolite discovery.

## 2. Materials and Methods

### 2.1. Strains and Culture Conditions

*A. cristatus* strain CM1303 was isolated from FBT (Jingyang, China). The strain was grown on high-concentration sodium chloride MYA solid medium (malt extract 20 g, yeast extract powder 5 g, agar powder 15 g, sucrose 30 g, NaCl 170 g, and water 1000 mL) at 37 °C to induce asexual spore production [9,21]. Potato glucose broth medium (PDB) liquid medium was used for RNA extraction. Induction medium (K_2_HPO_4_ 2.05 g, KH_2_PO_4_ 1.45 g, (NH_4_)_2_SO_4_ 0.5 g, MgSO_4_·7H_2_O 0.5 g, NaCl 0.15 g, CaCl_2_ 0.066 g, FeSO_4_·7H_2_O 0.00248 g, glucose 1.8 g, glycerol 5 mL, and distilled water to a constant volume of 940 mL) was used for *Agrobacterium* transformation. *Escherichia coli* Stbl3 competent cells and *Agrobacterium tumefaciens* AGL1 competent cells were purchased from Yuanye Boitech Co., Ltd. (Wuhan, China) and were cultured in LB liquid medium or grown on solid medium.

### 2.2. Cloning and Overexpression of the llm1 Gene

Total RNA was extracted using an Eastep ^®^ Super Total RNA Extraction Kit (Promega Biotech Co., Ltd., Beijing, China) and cDNA was prepared with FastKing gDNA Dispelling RT SuperMix (Tiangen Biotech Co., Ltd., Beijing, China). The overexpression vector pCAMBIA1303-TrpC-Hygro-gpdA-GFP (Fenghui Biotech Co., Ltd., Changsha, China), which mainly contains a constitutive promoter gpdA from *A. nidulans*, resistant kanamycin gene, and resistant hygromycin gene as selection markers, was linearized with restriction endonucleases *Bgl* II and *BstE* II. Primers Llm1F and Llm1R (Supplementary Table S1) were designed for amplification of *llm1* using the cDNA as a template and the instructions of the seamless cloning kit (Vazyme Biotech Co., Ltd., Nanjing, China). The gpdA-*llm1*-Trpc overexpression cassette was then constructed and the plasmid was confirmed by PCR, digestion and sequencing.

### 2.3. Fungal Transformation

Competent *A. tumefaciens* AGL1 containing the *llm1*-overexpression vector were inoculated into LB liquid medium and cultured for 48 h at 28 °C with a shaking speed of 180 rpm. The bacteria were collected by centrifugation, resuspended using IM induction liquid medium, diluted to an OD_600_ of 0.2, and cultured to an OD_600_ of 0.7. Next, 100 μL *Agrobacterium* solution and 100 μL *A. cristatus* spore solution (1 × 10^6^ conidia/mL) were mixed and spread on IM solid medium containing cellophane. After incubation at 28 °C for 2 d in the dark, the cellophane membrane was transferred to the primary screening medium (PDA containing 50 μg/mL hygromycin B and 200 μg/mL cefotaxime sodium) and suspected transformants were picked onto the rescreening medium (PDA containing 75 μg/mL hygromycin B). After culture for 7 d, RNA was extracted from the transformants and used to obtain cDNA, and the positive clones were verified by PCR amplification using HygroF and HygroR hygromycin fragment primers (Supplementary Table S1).

### 2.4. Phenotypic Analysis

The conidia (1 × 10^6^ conidia/mL) of *A. cristatus* wild-type (WT) and overexpression transformant (OE::*llm1*) were inoculated onto high-concentration NaCl MYA solid medium or MYA solid medium to observe the asexual and sexual development colony morphology, respectively. For further analysis, agar blocks (1 cm in diameter from the same location in the center of the surface of each sample) were collected, homogenized thoroughly by vortexing in a 1.5 mL centrifuge tube containing 1 mL sterile water, and spores or cleistothecia were counted using a hemocytometer. Oxidative stress resistance was determined by measuring the colony diameter after inoculating 15 μL conidia suspension (1 × 10^6^ conidia/mL) of the WT or OE::*llm1* transformant on PDA plates containing different concentrations of H_2_O_2_, culturing at 28 °C for 10 d. Resistance to oxidative stress was then expressed by the relative diameter size of the colonies.

### 2.5. LC–MS-Based Metabolite Profiling

A quantity of 3 mL conidia (1 × 10^6^ conidia/mL) of either the WT or OE::*llm1* transformant was inoculated into PDB (150 mL) and cultured on a shaker at 180 rpm and 28 °C for 7 d, and mycelium was collected by filtration. A quantity of 50 mg WT or OE::*llm1* transformant sample was taken, and 400 μL methanol: water = 4:1 (containing 0.02 mg/mL of internal standard L-2-chloro-phenylalanine) was added. After 6 min of grinding in a frozen tissue grinder and 30 min of low-temperature ultrasonic extraction (5 °C, 40 KHz), the sample was allowed to stand at −20 °C for 30 min. After centrifugation for 15 min (13,000× *g*, 4 °C), the supernatant was collected for metabolomics analysis.

Untargeted metabolomics analysis was performed using UHPLC-Triple TOF tandem time of flight mass spectrometry (AB SCIE X, Framingham, MA, USA). Chromatographic separation was carried out at 40 °C on an ACQUITY UPLC HSS T3 column (2.1 mm × 100 mm, 1.7 mum; Waters, Milford, MA, USA) with a binary gradient at a flow rate of 0.4 mL/min. Mobile phases A and B were formic acid aqueous solution (0.1%, *v/v*) and acetonitrile (containing 0.1% formic acid), respectively. The gradient procedure was as follows: 100% A (0–0.5 min), 100–75% A (0.5–2.5 min), 0% A (2.5–13 min), 0–100% A (13.0–13.1 min), and 100% A (13.1–16 min). Full scans and tandem MS acquisitions were performed in both negative and positive modes using the following parameters: full scans were recorded in the mass range m/z 50 to 1000 with a cycle time 510 ms; ion spray voltage, +5.0 kV for the positive ion mode and −4.0 kV for the negative ion mode; curtain gas, 35 PSI; ion source gas 1, 50 PSI; ion source gas 2, 50 PSI; and source temperature, 550 °C.

The metabolomics software, Progenesis QI (version 2.2), was used to process the raw data, and finally a data matrix containing information such as retention time, mass/charge ratio, and peak intensity was obtained. The MS and MS/MS mass spectra were matched with the metabolic database with the MS mass error set to less than 10 ppm, and the metabolites were identified based on the secondary mass spectra matching score. The Human Metabolome Database (HMDB, http://www.hmdb.ca/, accessed on 23 April 2022), METLIN database (https://metlin.scripps.edu/, accessed on 23 April 2022), and self-built databases of the Majorbio I-Sanger Cloud Platform (https://www.i-sanger.com, accessed on 23 April 2022) were used for retrieval. Unsupervised principal component analysis (PCA) and supervised partial least squares discrimination analysis (PLS-DA) were performed using the ropls package in R. According to the variable importance projection (VIP) value of the PLS-DA model, VIP > 1, *p* < 0.05, and fold change (FC) ≥ 1.5 or FC ≤ 0.67 were selected to find the differential metabolites.

### 2.6. Transcriptome Data Analysis

Conidia (1 × 10^6^ conidia/mL) from either the WT or OE::*llm1* transformant were inoculated into PDB liquid medium with 10% volume of inoculum, cultured at 30 °C (180 rpm/min) for 7 d, and mycelium was collected by filtration and rinsed with sterile water, and quickly ground into powder using liquid nitrogen. Total RNA was extracted using a TRIzol^®^ Reagent kit (Invitrogen, Carlsbad, CA, USA), and the quality and concentration of the extracted RNA were detected using a NanoDrop 2000 spectrophotometer (NanoDrop Technologies, Wilmingtom, DE, USA). Oligo (dT) primers and polyA of mRNA were used for complementary A-T pairing to isolate total mRNA, and a library was constructed using the Illumina TruseqTM RNA sample prep Kit method, which was used after quality inspection was passed, and the sequencing work was completed using an Illumina platform. Clean reads were obtained by quality control of the raw reads and the resulting reads were then mapped against predicted transcripts from the *Aspergillus cristatus* (GCA001717485.1) genome. The expression levels of genes and transcripts were analyzed using RSEM (http://deweylab.github.io/RSEM/, accessed on 2 May 2022), and the quantitative index was TPM (number of transcripts per million clean tags). Genes with significantly different expression levels (differentially expressed genes, DEGs) were identified by a significance test with combined thresholds (*p*-adjust < 0.05 and log2 FC ≥ 1). The raw RNA-Seq reads from WT and OE::*llm1* transformant have been deposited in the NCBI SRA database with the accession number PRJNA774889.

### 2.7. RT-qPCR Detection

After cDNA was obtained from WT and OE::*llm1* transformant, RT-qPCR was performed on the Applied Biosystems QuantStudio3 Real-Time PCR system (Thermo Biotech Co., Ltd., Waltham, MA, USA) according to the instructions of the SYBR Green PCR MasterMix (GeneStar Co., Ltd., Beijing, China), using *GAPDH* (glyceraldehyde-3-phosphate dehydrogenase) as the reference gene. Primers used for qPCR are shown in Supplementary Table S1. The relative transcription level of each tested gene was calculated using the 2^-ΔΔct^ method. All real-time PCR assays were performed in triplicate in each culture and repeated three times using RNA isolated from independent cultures.

### 2.8. Statistical Analysis

All data were the results of three repeated experiments and were statistically analyzed using GraphPad Prism version 8 (GraphPad Software, San Diego, CA, USA), and then the *t*-test was used for statistical analysis.

## 3. Results

### 3.1. Cloning and Analysis of the Gene llm1 in A. cristatus

**Figure 1:**
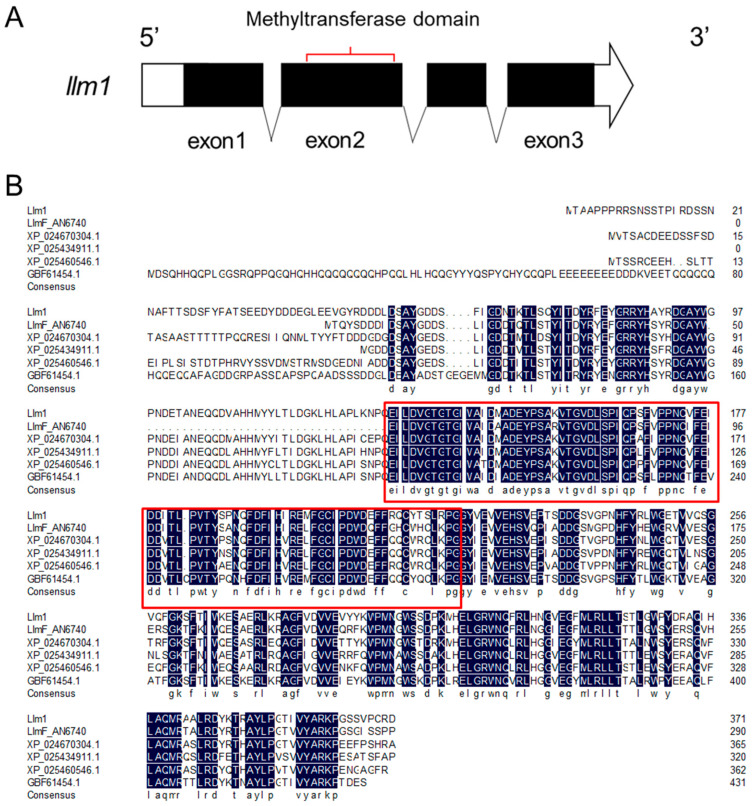
Bioinformatics analysis of *llm1*. (**A**) Structure diagram of *llm1* gene. (**B**) Alignment of the amino acid sequence of Llm1 with the sequences of *A*. *nidulans* (LlmF); *A*. *candidus* (XP_024670304.1); *A*. *saccharolyticus* (XP_025434911.1); *A. niger* (XP_025460546.1); and *Trichophyton mentagrophytes* (GBF61454.1). The navy boxes represent 100% conservatism and the red boxes indicate the location of the methyltransferase domain.

The *laeA*-like gene (*llm1*) was identified via Local-blast, using the *llmF* gene (*A. nidulans*, AN6749) as a query. The *llm1* gene was 1286 bp in length and the open reading frame (ORF) consisted of 1116 nucleotides, including three introns, which mapped to 241–296 bp, 736–805 bp, and 950–1000 bp, respectively. The gene encodes 371 amino acids and has a conserved S-adenosylmethionine-dependent methyltransferase domain at residues 133–221 (Figure 1A). BLASTP analysis revealed that the Llm1 protein sequence (ODM14516) shared 97.84% similarity with a protein from *A*. *chevalieri* (XP043139667.1) and 91.89% sequence similarity with a hypothetical protein from *A. glaucus* (XP_022397524.1). Multiple sequence alignments were performed between Llm1 and other *laeA*-like protein using DNAMAN and the result revealed that the N-terminus of Llm1 protein was not conserved and exhibited a very conservative methyltransferase domain sequence (Figure 1B).

### 3.2. Construction and Screening of llm1-Overexpressing Strain

The schematic diagram of *llm1* overexpression vector construction is shown in Supplementary Figure S1A. The putative *A. cristatus* transformants selected on hygromycin B-resistance PDA plates were chosen and grown for three generations. Finally, two transformants were screened out; the resulting two transformants could grow on PDA plates containing a high concentration of hygromycin B, whereas the WT could not (Supplementary Figure S1B). Simultaneously, RNA was extracted from the WT strain and transformants, and after obtaining cDNA, the hygromycin resistance fragment carried by the vector was amplified by PCR using HygroF and HygroR primers. Agarose gel electrophoresis of the resulting PCR products showed no corresponding band in the WT strain, whereas the transformants showed the corresponding fragment (406 bp) had been amplified (Supplementary Figure S1C), and the PCR products were verified by sequencing. QLlm1F and QLlm1R primers were used to validate the relative expression of the *llm1* gene (Supplementary Table S1), and the two transformants had significantly higher *llm1* expression than the WT strain (Supplementary Figure S1D), indicating the two transformants were desired transformants.

### 3.3. Effects of Overexpression of llm1 on Sex Development

**Figure 2:**
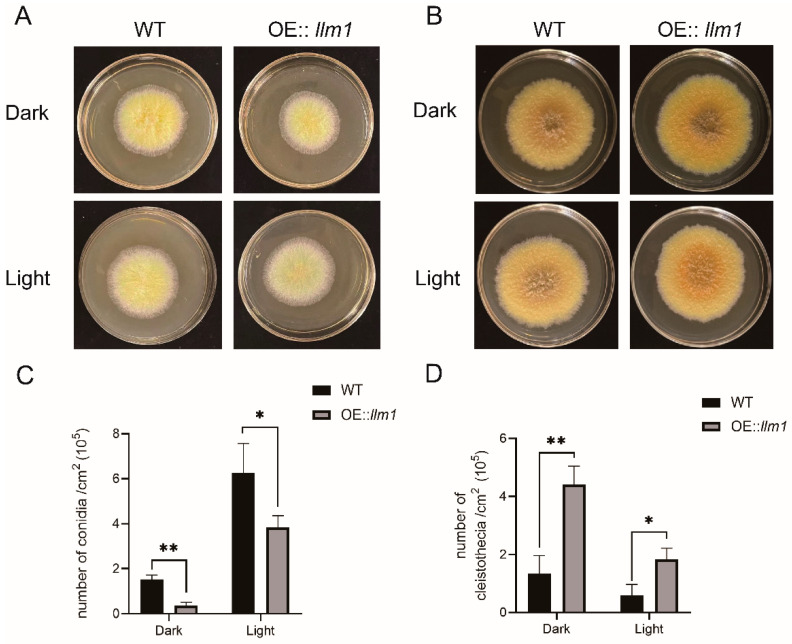
Effect of overexpression of *llm1* on sex development of *A. cristatus*. (**A**) Colony morphology of WT and OE::*llm1* transformant on MYA plate with high sodium chloride concentration. (**B**) Colony morphology of WT and OE::*llm1* transformant on MYA plate. (**C**) Statistics of conidia number produced by WT and OE: *llm1* transformant. (**D**) Statistics of cleistothecia number produced by WT and OE: *llm1* transformant. Experiments were conducted in three replicates. Standard deviations are indicated with error bars. Values that differed significantly from the value for the WT according to the *t*-test are indicated with asterisks (* *p* < 0.05, ** *p* < 0.01).

To investigate the effect of *llm1* gene overexpression on sex development of *A. cristatus*, the WT and OE::*llm1* strain were cultured in high-concentration sodium chloride MYA medium at 37 °C for 10 d. As shown in Figure 2A, compared to the WT, the OE::*llm1* transformant showed developmental retardation in both light and dark culture conditions. The production of conidia was markedly lower in the OE::*llm1* transformant (0.4 ± 0.1 × 10^5^ conidia/cm^2^) than in WT (1.5 ± 0.2 × 10^5^ conidia/cm^2^) in dark culture conditions and the same results were observed in light culture conditions (Figure 2C). However, we did not observe any obvious effect on colony morphological development during sexual development of *A. cristatus* (Figure 2B). Interestingly, the production of cleistothecia was markedly increased in the OE::*llm1* transformant (4.41 ± 0.51 × 10^5^ cleistothecia/cm^2^) compared to the WT (1.33 ± 0.51 × 10^5^ cleistothecia/cm^2^) in dark culture conditions and the results were very similar in light culture conditions (Figure 2D). These results suggest that overexpression of *llm1* appears to inhibit asexual development and enhance sexual development in *A. cristatus*.

### 3.4. Overexpression of the Gene llm1 Decreased Oxidative Stress Tolerance of A. cristatus

**Figure 3:**
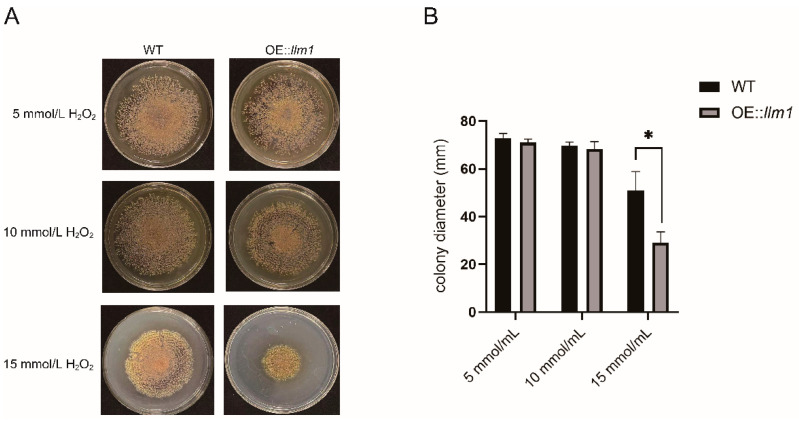
Phenotypes of WT and OE::*llm1* transformant under different concentrations of hydrogen peroxide. Growth (**A**) and colony diameter (**B**) were measured under different concentrations of hydrogen peroxide. Experiments were conducted in three replicates. Standard deviations are indicated with error bars. Values that differed significantly from the value for the WT according to the *t*-test are indicated with asterisks (* *p* < 0.05).

Putative methyltransferases are involved in the regulation of the tolerance of fungi to oxidative stress. Therefore, WT or the OE::*llm1* transformant was inoculated on PDA plates containing different concentrations of H_2_O_2_ (Figure 3A). There was no significant difference in strain morphology and growth rate between 5 and 10 mmol/mL H_2_O_2_. However, when the concentration of H_2_O_2_ was increased to 15 mmol/mL, the colony diameter of the OE::*llm1* transformant (28.9 ± 3.8 mm) was significantly lower than that of the WT strain (50.9 ± 6.6 mm) (Figure 3B). This means that the tolerance to oxidative stress was decreased in the overexpression transformant.

### 3.5. Effects of llm1 Overexpression on Metabolites Production

**Figure 4:**
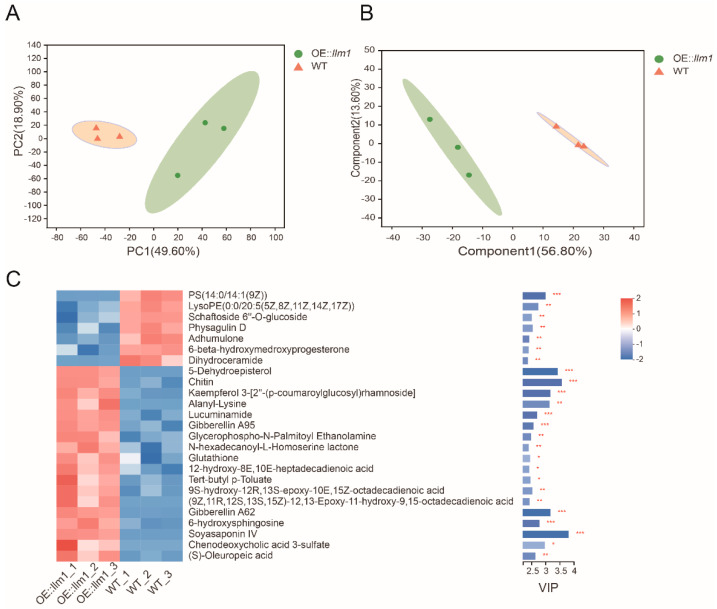
The metabolites in the OE::*llm1* group compared to those in the WT group. (**A**) PCA score plot of metabolite profiles from the OE::*llm1* group and WT group. (**B**) PLS-DA score plot of metabolite profiles from the OE::*llm1* group and WT group. (**C**) Heat map of VIP score calculated by PLS-DA. The color represents the relative abundance of the metabolites in the samples. On the right is the VIP bar graph of metabolites. The length of the bar represents the contribution value of the metabolite to the difference between the groups (* *p* < 0.05, ** *p* < 0.01, *** *p* < 0.001).

Studies have demonstrated that the putative methyltransferases represented by *laeA* are involved in a variety of life activities of filamentous fungi. Consequently, we used non-targeted metabolomics techniques to evaluate the effect of *llm1* overexpression on metabolism of *A. cristatus*. A total of 1238 effective metabolites were identified by searching a self-built database, METLIN database, and HMDB database. Inter-group differences and intra-group sample duplications were evaluated via PCA. As shown in Figure 4A, the first two principal components (PC1 and PC2) can clearly separate the two groups of samples, which explains the changes of 49.60% and 18.90%, respectively, indicating significant differences in metabolites between the two groups. The PLS-DA model was used to screen differential compounds between the two groups, as shown in the PLS-DA chart (Figure 4B). WT samples were mainly distributed on the left side of the confidence interval, and the OE:: *llm1* groups were mainly distributed on the right side, indicating that the model can effectively distinguish the metabolites of the two groups. 

Based on the PLS-DA model, a total of 80 different metabolites (VIP > 1, *p* < 0.05 and FC ≥ 1.5 or FC ≤ 0.67) were selected (Supplementary Table S2). The HMDB database classification annotation results showed that different metabolites were primarily involved in lipids and lipid-like molecules (28 species, 35%), organic oxygen compounds (9 species, 11.25%), organic acids and derivatives (6 species, 7.5%), organoheterocyclic compounds (4 species, 5%) and phenylpropanoids and polyketides (3 species, 3.75%). The top 25 important compounds with VIP scores and their relative abundances were shown by heat map (Figure 4C). The relative contents of 18 metabolites were significantly increased, including terpenoids ((S)-oleuropeic acid, soyasaponin IV, gibberellin A62, gibberellin A95), steroids ((5-dehydroepisterol), kaempferol 3-[2′-(p-coumaroylglucosyl) rhamnoside]) and two long-chain unsaturated fatty acids (9s-hydroxy-12r,13s-epoxy-10e,15z octadecadienoic acid and (9z,11R,12s,13s,15z)-12,13-epoxy-11-hydroxy-9,15-octadecadienoic acid). A total of 7 metabolites were significantly reduced, including two glycerol phospholipids (PS(14:0/14:1(9Z)) and lysoPE (0:0/20:5(5Z,8Z,11Z,14Z,17Z))), two kinds of steroid compounds (physagulin D and 6-beta–hydroxymedroxyprogesterone) and a kind of flavonoid compound (schaftoside 6′-O-glucoside). Notably, we found that a variety of typical secondary metabolites were also noted in the 80 important differential compounds, mainly including flavonoids (ovalitenone, PD 98059, 1-isomangostin, tricin 7-[Feruloyl-(->2)-glucuronyl-(1->2)-glucuronide] and kaempferol 3–[2′-(p-coumaroylglucosyl) rhamnoside]), terpenoids (alpha-santalyl acetate, avenestergenin A1, (+/−)-2,4,8-trimethyl-7-nonen-2-ol, 2beta,9xi-dihydroxy-8-oxo-1(10),4,11(13)-germacratrien-12,6alpha-olide), and a variety of organic acids. The results showed that overexpression of *llm1* enhanced the production of secondary metabolites represented by terpenoids and flavonoids in *A. cristatus.*

### 3.6. Transcriptome Data Analysis

After quality control of raw reads, the number of clean reads obtained was greater than 5 million for each different sample. In addition, the average error rate of sequencing bases corresponding to the quality control data of each sample was below 0.1%, and GC content was around 54%, indicating the quality of the data was excellent (Supplementary Table S3). Gene expression was quantified in each sample, then Pearson correlation analysis was further performed on three repeats of each sample (WT group and OE::*llm1* group) to evaluate the reproducibility of the experiment. The correlation between samples was very high (Supplementary Figure S2), indicating that the sequencing results were reliable.

**Figure 5:**
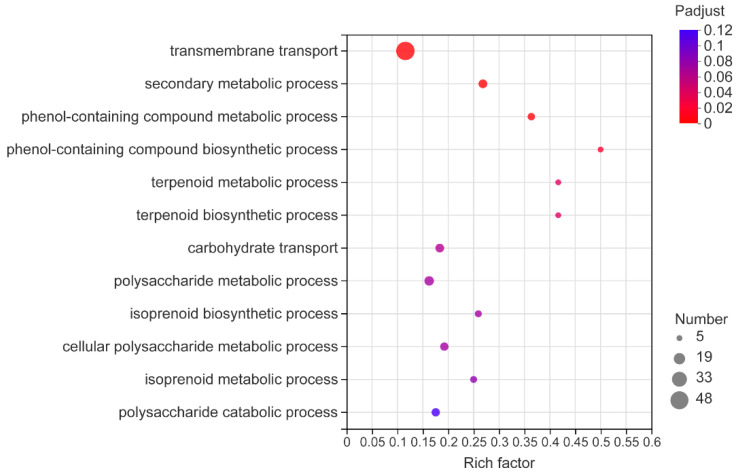
Enriched biological process Gene Ontology (GO) terms in DEGs.

A total of 10,344 genes were identified by transcriptome sequencing, of which 600 genes were significantly differentially expressed with 246 genes being upregulated and 354 genes being downregulated (Supplementary Table S4). The Gene Ontology (GO) database was used for functional enrichment analysis of the DEGs, and the DEGs were enriched in 130 functional pathways. Among them, biological process (BP) accounted for 72/130, cellular component (CC) accounted for 3/130, and molecular function (MF) accounted for 55/130. Our research was mainly focused on biological process (BP), the DEGs were mainly involved in transport and secondary metabolism-related biological processes (Figure 5), and the most significant enrichment pathway was transmembrane transport (GO: 0055085), accounting for 10.58% of the DEGs in BP. Secondary metabolic process (GO: 0019748), phenol-containing compound metabolic process (GO: 0018958), secondary metabolite biosynthetic process (GO: 0044550), phenol-containing compound biosynthetic process (GO: 0046189), isoprenoid biosynthetic process (GO: 0008299), and terpenoid metabolic process (GO: 0006721), accounted for 10.59% of the DEGs in BP, indicating that most of the DEGs were enriched in pathways related to secondary metabolites. Interestingly, consistent with the results obtained from metabonomics, we found that there were significant differences in the expression of numerous genes in the terpenoid biosynthetic process, mainly including farnesyl diphosphate syntheses (SI65_08119 and SI65_09732), acetyltransferase (SI65_03723), and isopentenyl diphosphate delta isomerase (SI65_05138).

### 3.7. Expression of Secondary Metabolite-Related Gene Clusters

To better understand the regulation of *llm1* overexpression on secondary metabolite of *A. cristatus,* secondary metabolite biosynthetic gene clusters were re-predicted by antiSMASH based on genome and transcriptome data [22]. A total of 37 secondary metabolite gene clusters were predicted, comprising a total of 582 related genes. The DEGs were analyzed and 64 genes with significant differences in expression were obtained (Supplementary Table S5), accounting for 11.00% of the predicted secondary metabolite-related genes. The core genes in each gene cluster were annotated, and their expression levels were further analyzed (Table 1). Interestingly, there were 11 significantly upregulated core genes, including non-ribosomal peptide synthetase (SI65_08405, SI65_05234, and SI65_07252), a polyketide (SI65_03722), and two genes annotated as Cytochrome P450 monooxygenase (SI65_05770 and SI65_05771). Five core genes were significantly downregulated and belonged to different gene clusters, with three of them predicted to encode polyketide synthase (SI65_09735, SI65_03276, and SI65_06673). These results further indicated that overexpression of *llm1* activated the expression of gene clusters of secondary metabolites in *A. cristatus*.

The accuracy of the transcriptome data was verified by analyzing the expression of seven secondary metabolite genes, including four upregulated genes (SI65_03722, SI65_05768, S165-08405, and SI65_05769) and three downregulated genes (SI65_09735, SI65_01982, and SI65_06672). The transcriptome data and RT-qPCR results were consistent (Supplementary Figure S3).

## 4. Discussion

The putative methyltransferases represented by *laeA* are thought to play an important role in the regulation of fungal sex development, secondary metabolism, and oxidative stress [23,24,25]. In *A.* *nidulans*, LaeA can form heterotrimers with the velvet family proteins VeA and VelB in the nucleus, thereby activating sex development and regulation of secondary metabolite gene clusters [26]. LlmF, a methyltransferase similar to LaeA, was involved in the regulation of secondary metabolites in *A. nidulans*, and yeast two-hybrid experiments showed that LlmF and LaeA were similar and could interact with VeA, thus participating in the regulation of secondary metabolites [17,27]. A *laeA*-like methyltransferase in *Penicillium oxalicum* also had a regulatory role in sex development and secondary metabolism, but there was no evidence that this protein can interact with the VeA [28]. *llmF* [17], *llm1* [18], and *lael* 1 [29] were thought to negatively regulate the synthesis of secondary metabolites. However, *llmG* seemed to exhibit a positive regulatory influence on the regulation of secondary metabolites [19]. These observations illustrate that the regulation of secondary metabolites by putative methyltransferases in filamentous fungi was diverse.

This paper reports the effects of the overexpression of a putative methyltransferase (*llm1*) on morphological development and secondary metabolism in *A. cristatus*. The results from the current study indicated that *llm1* may be a negative regulator of oxidative tolerance in *A. cristatus*. However, in *C. heterostrophus*, a putative methyltransferase gene (*llm1*) is not considered to be involved in the regulation of oxidative stress processes [18]. This indicates that *laeA*-like methyltransferases from different sources have different functions in the regulation of fungal oxidative tolerance. Secondary metabolites are instrumental in the defense against oxidative stress [30], so we speculate that the regulation of *llm1* on oxidative tolerance may be related to the activation of secondary metabolite gene clusters.

Overexpression of *llm1* resulted in a significant decrease in conidia and increase in cleistothecia; this regulation was not affected by light. Similarly, *veA* was not affected by light in the regulation of sex development of *A. cristatus*, which is different from other members of *Aspergillus* spp. [31]. Among a few studies on *laeA*-like methyltransferases, overexpression of the gene *llmF* in *A.* *nidulans* resulted in the inhibition of sexual development [17], and similar results were reported in *C. heterostrophus* [18]. This is contrary to the conclusion from the current study and suggested that *laeA*-like methyltransferases from different sources may also be involved in the regulation of fungal development while their effects may be different.

Interestingly, studies have demonstrated that genetic regulators (e.g., *veA* and *acndtA*) controlling the sex development in *A. cristatus*, also govern the production of secondary metabolites [21,31]. Based on this view, we further used metabolomics and transcriptomics to evaluate the effect of *llm1* overexpression on secondary metabolites of *A. cristatus*. UHPLC-QTOF-MS/MS revealed a differential metabolic profile with 80 metabolites that were significantly different. Notably, the contents of several secondary metabolites, including terpenoids, flavonoids, and steroids, increased significantly. The same result was confirmed in transcriptome sequencing, with a total of 600 DEGs in the OE::*llm1* transformant compared to WT. There were several terms related to secondary metabolism in GO enrichment analysis of DEGs, mainly including terpenoids and phenol-containing compound biosynthetic process. This further illustrated the regulatory effect of *llm1* on secondary metabolites of *A. cristatus*. These results were consistent with transcriptome data obtained after the *laeA* gene was knocked out in *Valsa mali* [32]. Expression analysis was additionally performed for secondary metabolism-related genes, and significant differences were found for 11.00% of the genes. Notably, the expression levels of core enzymes in multiple secondary metabolite gene clusters were significantly upregulated, mainly including non-ribosomal peptide synthetase, polyketide, and cytochrome P450 monooxygenase, which were thought to play an important role in the synthesis and modification of secondary metabolite [33,34,35,36]. Overexpression of the gene *laeA* in *A. niger* also resulted in differential expression of numerous secondary metabolite genes [37], suggesting that *llm1* may resemble *laeA* as a global regulator gene that controls the expression of secondary metabolite gene clusters. The strategy of activating secondary metabolic silencing gene clusters in filamentous fungi has shown that the combined editing of multiple genes is often more efficient than knocking out or overexpressing a single gene [19], which provides additional ideas for future in-depth exploration of secondary metabolites of *A. cristatus*. 

## 5. Conclusions

In this study, we characterized a putative methyltransferase (*llm1*) from *A. cristatus* and demonstrated that overexpressing the *llm1* gene can effectively activate the silencing gene cluster of secondary metabolites of *A. cristatus* and increase the production of secondary metabolites. Our study provides new insights and research means for the discovery of new secondary metabolites produced by *A. cristatus*.

## Figures and Tables

**Table 1:** Expression analysis and functional annotation of core genes in secondary metabolic gene clusters.

	Accession	Description	Cluster
**Up-regulated**	SI65_08318	Acyl-CoA ligase	8
	SI65_08405	Non-ribosomal peptide synthetase	9
	SI65_09733	Cytochrome P450 monooxygenase	17
	SI65_10096	Diacylglycerol lipase	21
	SI65_03722	Polyketide synthase	24
	SI65_05234	Non-ribosomal peptide synthetase	30
	SI65_05768	Atrochrysone carboxylic acid synthase	32
	SI65_05770	Cytochrome P450 monooxygenase	32
	SI65_05771	Cytochrome P450 monooxygenase	32
	SI65_05772	Oxidoreductase	32
	SI65_07252	Non-ribosomal peptide synthetase	36
**Down-regulated**	SI65_09735	Polyketide synthase	17
	SI65_03276	Polyketide synthase	23
	SI65_05416	Non-ribosomal peptide synthetase	31
	SI65_06672	Cytochrome P450 monooxygenase	34
	SI65_06673	Polyketide synthase	34

## Data Availability

Data used to support the findings of this study are available from the corresponding author upon request.

## Supplementary Materials

The following supporting information can be downloaded at: https://www.mdpi.com/article/10.3390/microorganisms10091707/s1, Figure S1: Construction of overexpression vector and identification of overexpressing *llm1* transformants. (A) Construction of the *llm1*-overexpression vector. (B) Growth of WT and two transformants under high concentrations of hygromycin. (C) Amplification of hygromycin gene resistance fragment in transformants. Lane M: molecular weight standard (DNA Marker 2000), lane 5: positive control, lane 4: negative control, lane 3: wild-type, lanes 1–2: two transformants. (D) Relative expression level of *llm1* in two transformants. Experiments were conducted in three replicates. Standard deviations are indicated with error bars. Values that differed significantly from the value for the WT according to the *t*-test are indicated with asterisks (* *p* < 0.05, ** *p* < 0.01). Figure S2: Correlation heat map of samples; Figure S3: RT-qPCR verification of WT and OE::*llm1* transformants; Table S1: Primers used in this study; Table S2: Differentially expressed metabolites between OE::*llm1* and WT group; Table S3: Mapping statistics of RNA-seq data; Table S4: Differentially expressed genes between OE::*llm1* and WT group; Table S5: Significantly differentially expressed secondary metabolite synthesis genes screened based on transcriptome data and antiSMASH annotation.
