# Supplementary material for: Overexpression of llm1 Affects the Synthesis of Secondary Metabolites of Aspergillus cristatus
Source: Microorganisms. 2022 Aug 24;10(9):1707. doi: 10.3390/microorganisms10091707 (PMC9502445; doi:10.3390/microorganisms10091707)
Supplement: Supplementary file 1 [file microorganisms-10-01707-s001.zip › Support Figures.pdf]

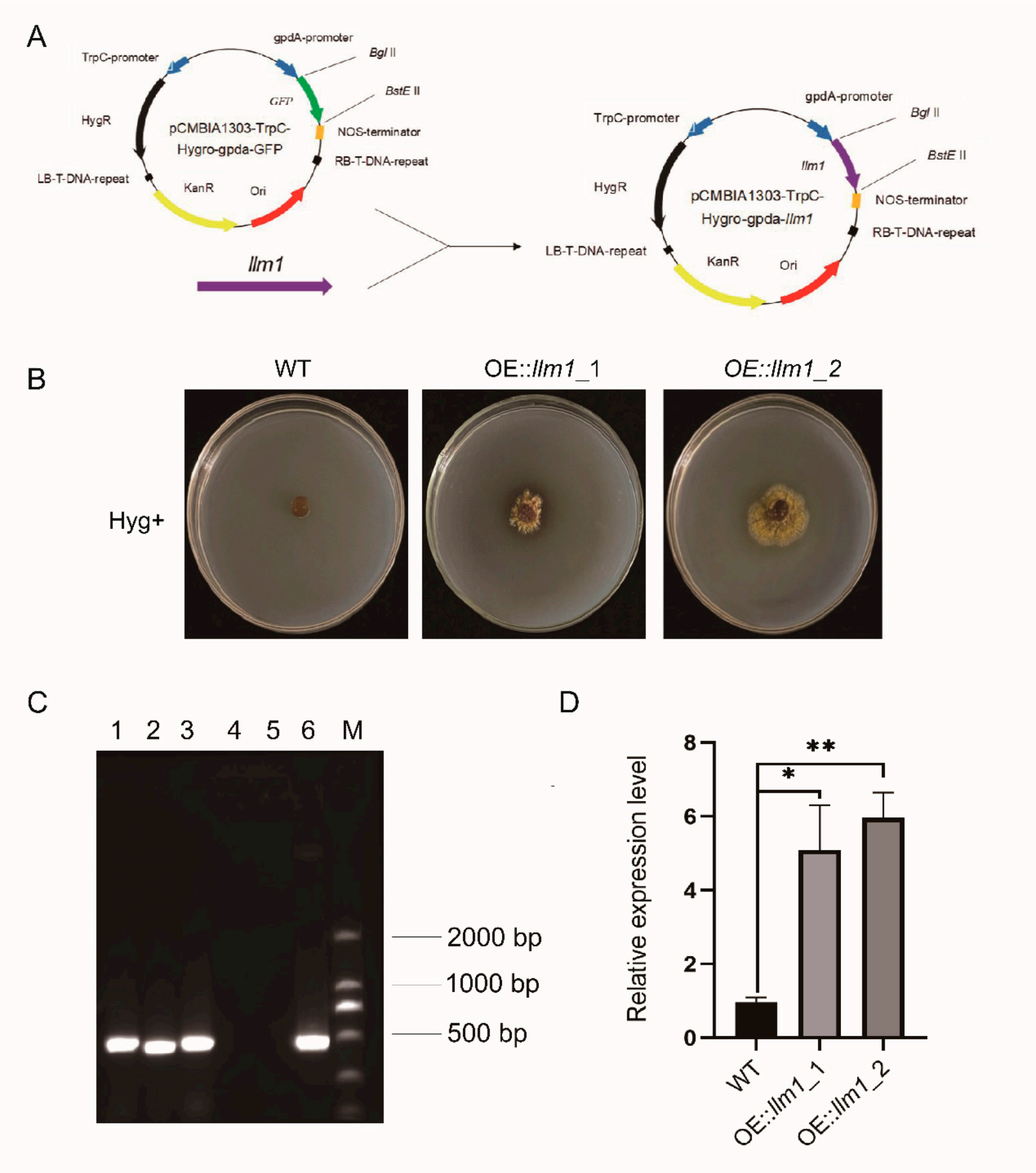

Figure S1 Construction of overexpression vector and identification of overexpressing *llm1* mutant. (A) Construction of the *llm1*-overexpression vector. (B) Growth of WT and two transformants under high concentrations of hygromycin. (C) Amplification of Hygromycin gene resistance fragment in transformants. Lane M: molecular weight standard (DNA Marker 2000), lane 5: positive control, lane 4: negative control, lane 3: wild-type, lanes 1-2: two transformants. (D) Relative expression level of *llm1* in two transformants. Experiments were conducted in three replicates. Standard deviations are indicated with error bars. Values that differed significantly from the value for the WT according to the *t*-test are indicated with asterisks (*$p < 0.05$, ** $p < 0.01$).

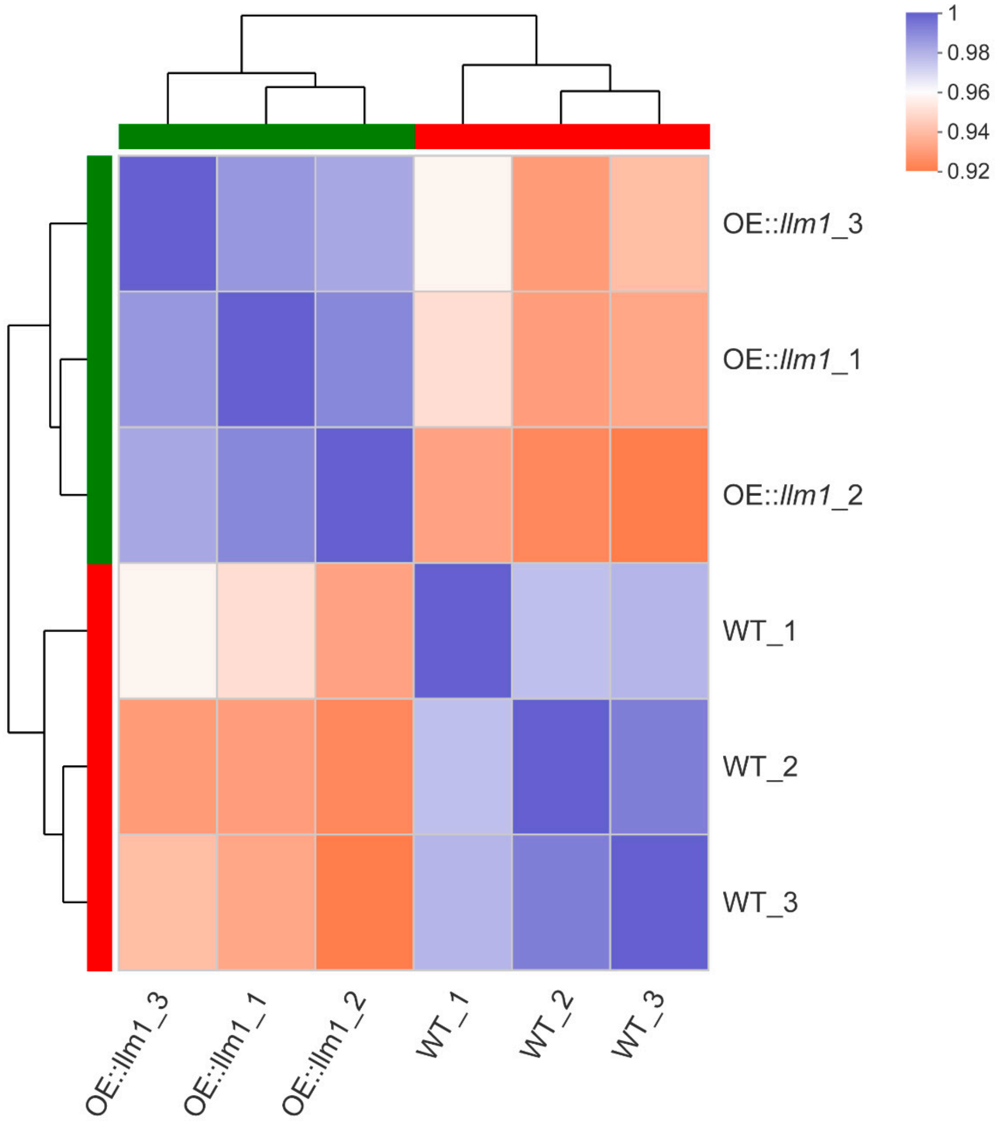

Figure S2 Correlation heat map of samples.

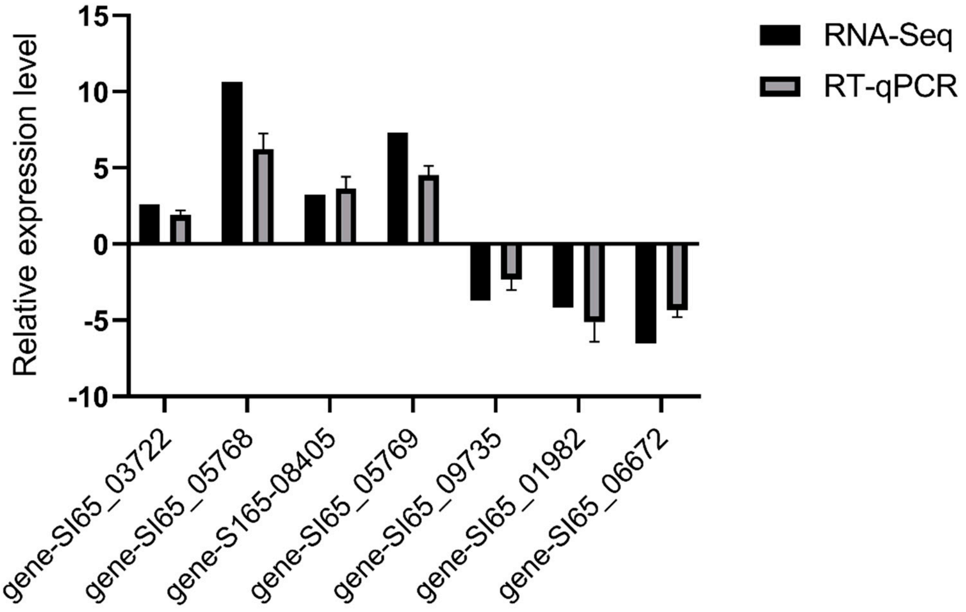

Figure S3 RT-qRCR validation of differentially expressed genes
