# Supplementary material for: Overexpression of llm1 Affects the Synthesis of Secondary Metabolites of Aspergillus cristatus
Source: Microorganisms. 2022 Aug 24;10(9):1707. doi: 10.3390/microorganisms10091707 (PMC9502445; doi:10.3390/microorganisms10091707)
Supplement: Supplementary file 1 [file microorganisms-10-01707-s001.zip › Table_S1.pdf]

Table S1 Primers used in this study

Primer	Sequence
Llm1F	agacatcaccatggtagatctCCTTGTGGCTACGGAGTGCA
Llm1R	ggggaaattcgagctggtcaccGCCGCCCGCAACTTAATC
HygroF	ATCGTTATGTTTATCGGCACT
HygroR	TGGCGACCTCGTATTGG
QLlm1F	AGTCCAAATCATCGTCCCG
QLlm1R	GCTCCAACTCCTCCACTCC
GAPDHF	CTGCCGTATCGAGAAGGGTG
GAPDHR	GATGAAGTTGGGGTTGAGGG
SI65_03722F	GGGAAGCGTATGAGAAGAT
SI65_03722R	ATTGAAAAGAACAGGGGAC
SI65_05768F	GATAGTTTGGCTGGATTGC
SI65_05768R	TCCTTACCCTTGGTGAGAG
SI65_08405F	TTTGAGTGGAACGAGGAGT
SI65_08405R	GGTGTCGGGTGTAGATGTC
SI65_05769F	GTGTGCTTGCGGGTAATCC
SI65_05769R	ATCGCCATGCCAGTGGGTG
SI65_09735F	TCTGTTTTATCGGTGTTTG
SI65_09735R	TGGTAGGAAAGATGGGCAG
SI65_01982F	CAAAAGCCATACAACCAGG
SI65_01982R	CACTAACTCCAATGACCCA
SI65_06672F	TTCAGCAAGAACGCAGCAG
SI65_06672R	CGTAACCCTAGCAAGACCG
