# Supplementary material for: Overexpression of llm1 Affects the Synthesis of Secondary Metabolites of Aspergillus cristatus
Source: Microorganisms. 2022 Aug 24;10(9):1707. doi: 10.3390/microorganisms10091707 (PMC9502445; doi:10.3390/microorganisms10091707)
Supplement: Supplementary file 1 [file microorganisms-10-01707-s001.zip › Table_S3.pdf]

Table S3 Mapping statistics of RNA-seq data

Sample	Raw reads	Clean reads	Clean Ratio	Q20(%)	Q30(%)	GC content(%)
OE::*llm1*_1	56132880	55763098	0.993412381	98.36	95.09	54.22
OE::*llm1*_2	63294932	62772572	0.991747207	98.4	95.18	54.32
OE::*llm1*_3	52481180	52067124	0.992110391	98.33	95.02	54.36
WT_1	52014002	51659080	0.993176414	98.41	95.24	54.22
WT_2	57594222	57246210	0.993957519	98.43	95.26	54.19
WT_3	58672768	58282756	0.993352759	98.42	95.25	54.26
