# Supplementary material for: Overexpression of llm1 Affects the Synthesis of Secondary Metabolites of Aspergillus cristatus
Source: Microorganisms. 2022 Aug 24;10(9):1707. doi: 10.3390/microorganisms10091707 (PMC9502445; doi:10.3390/microorganisms10091707)
Supplement: Supplementary file 1 [file microorganisms-10-01707-s001.zip › Table_S5.pdf]

Table S5 Significantly differentially expressed secondary metabolite synthesis genes screened based on transcriptome data and antiSMASH annotation.

Gene _ID	Cluster ID	LOG2 (FC)	*P*-adjust	Regulate
SI65_01982	Cluster4	-1.96	2.69e-10	down
SI65_08474	Cluster10	-2.82	6.04e-77	down
SI65_08573	Cluster11	-1.55	0.001033	down
SI65_02863	Cluster16	-1.46	0.000007	down
SI65_09734	Cluster17	-1.44	0.003393	down
SI65_09735	Cluster17	-1.79	3.87e-59	down
SI65_09798	Cluster18	-1.35	0.000002	down
SI65_09799	Cluster18	-2.23	1.59e-30	down
SI65_10009	Cluster20	-1.87	0.000016	down
SI65_10012	Cluster20	-4.32	0.000011	down
SI65_10092	Cluster21	-1.18	0.024799	down
SI65_03265	Cluster23	-2.08	7.64e-10	down
SI65_03266	Cluster23	-2.19	3.89e-11	down
SI65_03269	Cluster23	-2.15	1.59e-9	down
SI65_03270	Cluster23	-2.57	6.67e-20	down
SI65_03271	Cluster23	-1.53	1.05e-28	down
SI65_03272	Cluster23	-1.30	1.79e-23	down
SI65_03273	Cluster23	-1.41	9.85e-20	down
SI65_03275	Cluster23	-1.55	4.02e-22	down
SI65_03276	Cluster23	-2.89	0.006066	down
SI65_03776	Cluster25	-2.44	3.31e-66	down
SI65_05416	Cluster31	-1.40	0.023200	down
SI65_05422	Cluster31	-3.38	2.08e-18	down
SI65_06672	Cluster34	-2.61	4.39e-11	down
SI65_06673	Cluster34	-2.11	2.12e-37	down
SI65_06674	Cluster34	-2.44	4.13e-62	down
SI65_06675	Cluster34	-3.65	0.000876	down
SI65_07257	Cluster36	-3.64	1.38e-15	down
SI65_07680	Cluster5	2.19	9.11e-10	up
SI65_08206	Cluster7	1.47	8.84e-7	up
SI65_08207	Cluster7	1.98	3.16e-8	up
SI65_08318	Cluster8	1.79	6.17e-9	up
SI65_08405	Cluster9	1.82	3.42e-10	up
SI65_08578	Cluster11	1.82	0.000510	up
SI65_09732	Cluster17	1.82	1.85e-10	up
SI65_09733	Cluster17	1.25	5.89e-11	up
SI65_10002	Cluster20	5.46	0	up
SI65_10096	Cluster21	1.27	7.34e-12	up
SI65_03283	Cluster23	3.25	0	up
SI65_03722	Cluster24	1.48	5.51e-24	up
SI65_03723	Cluster24	1.86	1.12e-18	up

SI65_03724	Cluster24	1.20	3.27e-14	up
SI65_03725	Cluster24	1.76	2.96e-36	up
SI65_04502	Cluster27	1.40	1.51e-22	up
SI65_04506	Cluster27	1.91	5.53e-10	up
SI65_05234	Cluster30	1.28	1.90e-11	up
SI65_05762	Cluster32	1.26	3.17e-40	up
SI65_05764	Cluster32	1.98	5.55e-43	up
SI65_05767	Cluster32	2.01	1.08e-7	up
SI65_05768	Cluster32	3.51	5.976e-9	up
SI65_05769	Cluster32	2.97	1.70e-10	up
SI65_05770	Cluster32	3.03	1.31e-9	up
SI65_05771	Cluster32	3.49	8.70e-10	up
SI65_05772	Cluster32	3.51	1.51e-12	up
SI65_05773	Cluster32	4.21	2.32e-11	up
SI65_07248	Cluster36	1.25	5.46e-32	up
SI65_07249	Cluster36	1.12	8.33e-21	up
SI65_07250	Cluster36	2.22	7.45e-18	up
SI65_07251	Cluster36	1.38	1.53e-31	up
SI65_07252	Cluster36	1.28	4.92e-33	up
